# Jiawei Duhuo Jisheng Mixture Alleviates Knee Osteoarthritis via Inhibition of TLR4/MyD88/NF‐κB Signaling and Is Associated With Gut Microbiota Alterations

**DOI:** 10.1002/iid3.70586

**Published:** 2026-11-12

**Authors:** Jin Wu, Zhi Wen, Zifeng Ye, Liguo Qiu, Guang Xia, Xuyi Tan, Tikang Guo, Yong Tang, Min Lu, Xiaotong Xu, Gaoyan Kuang

**Affiliations:** ^1^ Department of Orthopedic Surgery The First Hospital of Hunan University of Chinese Medicine Changsha China; ^2^ Hunan University of Medicine General Hospital Huaihua China; ^3^ Affliated Hospital of Hunan Academy of Traditional Chinese Medicine Changsha China; ^4^ Shenzhen Institutes of Advanced Technology Chinese Academy of Sciences Shenzhen China

**Keywords:** cartilage degeneration, gut microbiota, inflammatory cytokines, Jiawei Duhuo Jisheng Mixture, knee osteoarthritis, TLR4/MyD88/NF‐κB

## Abstract

**Objective:**

To evaluate the chondroprotective effects of Jiawei Duhuo Jisheng Mixture (JDJM) in KOA and to determine whether JDJM modulates the TLR4/MyD88/NF‐κB axis and is associated with gut microbiota alterations.

**Methods:**

A KOA model was established in New Zealand rabbits using a modified Videman method (*n* = 36). Animals were assigned to a blank control group (*n* = 6) or underwent modeling and were then randomized into the model, JDJM low‐, medium‐, or high‐dose, and positive control groups (*n* = 6/group). Treatments were administered intragastrically for 6 weeks. Cartilage histopathology, evaluated by H&E staining and OARSI scoring, constituted the primary histopathological assessment, with the OARSI score serving as the quantitative primary endpoint. Secondary mechanistic outcomes included serum inflammatory cytokines and TLR4/MyD88/NF‐κB‐related measures in cartilage tissue, including NF‐κB p65 nuclear translocation. In vitro, primary rabbit chondrocytes were exposed to IL‐1β–induced inflammatory stimulation to provide supportive mechanistic evidence through assessment of cell viability, apoptosis, inflammatory mediators, and pathway‐related molecules. Molecular docking was additionally performed to explore potential interactions between identified JDJM constituents and TLR4/MD‐2, MyD88, and NF‐κB p65. Fecal 16S rRNA sequencing and related gut microbiota analyses were considered exploratory.

**Results:**

Compared with the model group, JDJM attenuated cartilage histopathological damage and reduced OARSI scores, while decreasing serum levels of pro‐inflammatory cytokines (IL‐1β, IL‐6, TNF‐α, and IFN‐γ). In cartilage tissue, JDJM reduced TLR4, MyD88, and NF‐κB expression and suppressed NF‐κB p65 nuclear translocation. In vitro, JDJM increased the viability of IL‐1β–stimulated chondrocytes, reduced apoptosis, and decreased the release of inflammatory mediators. Molecular docking revealed distinct docking profiles of JDJM constituents against TLR4/MD‐2, MyD88, and NF‐κB p65. 16S rRNA sequencing indicated a shift in gut microbiota composition in the KOA model. Following JDJM treatment, microbiota composition was modulated, and several bacterial genera showed significant correlations with inflammatory markers and pathway‐related molecules.

**Conclusion:**

JDJM exerted anti‐inflammatory and chondroprotective effects in in vivo KOA models and in vitro primary chondrocytes. These effects may be mediated, at least in part, by inhibition of the TLR4/MyD88/NF‐κB signaling axis. The observed microbiota changes suggest that the gut–joint axis may contribute to the overall effects, although causal relationships require further validation.

## Graphical abstract

Created in BioRender. Wu (2026) https://BioRender.com/sah20xd.

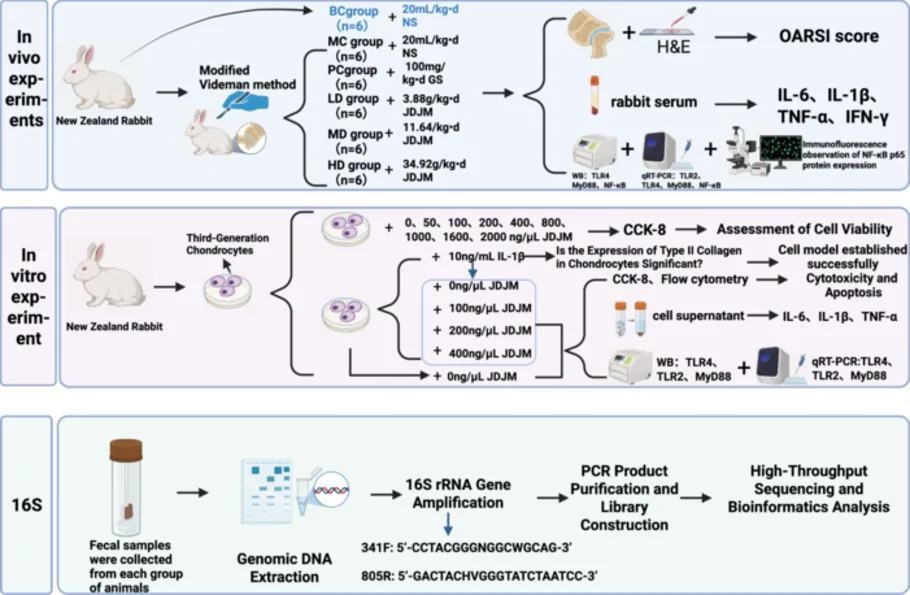

## 1 Introduction

Knee osteoarthritis (KOA) is a degenerative joint disease characterized by chronic pain and functional limitation that substantially reduces quality of life in middle‐aged and older adults [1, 2]. Core pathological features include progressive articular cartilage degeneration and structural changes, including subchondral bone remodeling and sclerosis and osteophyte formation; advanced disease may lead to joint deformity and persistent functional impairment [3]. Although joint replacement can improve function in end‐stage disease, its applicability is limited by surgical risk, postoperative complications, and cost [4]. Therefore, in early to mid‐stage KOA, relieving pain and inflammation while delaying structural deterioration remains a major therapeutic challenge [5]. KOA has increasingly been recognized as a whole‐joint disease involving multiple tissues, including cartilage, subchondral bone, synovium, and ligaments [6]. Low‐grade chronic inflammation and disrupted immune homeostasis are considered key drivers of progression. Synovitis and local cartilage inflammation can sustain joint microenvironmental dysfunction via pro‐inflammatory mediators, promote extracellular matrix degradation, exacerbate pain, and interact with structural changes, forming a vicious cycle of inflammation–degeneration–pain [7, 8].

Innate immunity–related signaling pathways play a key role in inflammatory amplification in KOA [9]. Damage‐associated molecular patterns (DAMPs) can trigger inflammatory cascades via pattern‐recognition receptors, and Toll‐like receptors (TLRs) represent critical nodes linking tissue damage, immune activation, and structural degeneration [10]. TLR4 is of particular interest because its activation can initiate signaling via adapter proteins such as myeloid differentiation factor 88 (MyD88), thereby activating nuclear factor‐κB (NF‐κB)–dependent transcription and increasing pro‐inflammatory cytokine expression, including interleukin‐1β (IL‐1β), tumor necrosis factor‐α (TNF‐α), and IL‐6; these changes can exacerbate synovitis and chondrocyte catabolic responses [11]. Notably, NF‐κB activation is reflected not only by changes in expression but also by nuclear translocation and increased transcriptional activity of key subunits such as NF‐κB p65 [12]. Therefore, targeting the TLR4/MyD88/NF‐κB axis may help suppress inflammatory amplification and slow structural progression in KOA.

Traditional Chinese medicine may offer advantages for degenerative joint diseases through holistic regulation and multitarget effects [13]. Jiawei Duhuo Jisheng Mixture (JDJM) is a hospital preparation from the First Affiliated Hospital of Hunan University of Chinese Medicine and has been used clinically. Our previous studies suggest that JDJM reduces KOA‐related inflammatory mediators and may improve joint inflammation and histopathological injury in animal models, consistent with modulation of inflammatory signaling pathways [14, 15, 16]. However, studies concurrently evaluating its anti‐inflammatory and chondroprotective effects in both in vivo models and primary‐cell in vitro systems remain limited. In particular, a coherent evidence chain for pathway activation—especially functional indices of NF‐κB activity—has not been established. In addition, potential systemic mechanisms of JDJM require further elucidation.

The gut–joint axis has been proposed as a framework for inflammation‐driven KOA [17]. Existing studies have reported differences in gut microbiota composition and metabolic profiles between KOA patients and controls. Microbiota dysbiosis may disrupt joint homeostasis by increasing intestinal permeability, promoting low‐grade systemic inflammation, and altering short‐chain fatty acid and tryptophan metabolism [18, 19]. When the intestinal barrier is compromised, gut‐derived lipopolysaccharide (LPS) can more readily enter the circulation. As a key TLR4 ligand, LPS may contribute to MyD88/NF‐κB–mediated inflammatory amplification [20]. Therefore, it remains unclear whether herbal formula intervention is accompanied by microbiota alterations and whether these changes are associated with joint inflammatory phenotypes and TLR4 signaling activation; more definitive experimental evidence is needed.

Based on these gaps, it was hypothesized that JDJM alleviates KOA‐related inflammation and exerts chondroprotective effects by inhibiting TLR4/MyD88/NF‐κB signaling and that these effects are accompanied by gut microbiota alterations. To test this hypothesis, a parallel in vivo and in vitro strategy was employed. In a rabbit KOA model, JDJM effects on joint histopathology and inflammatory phenotypes were evaluated, and expression of key TLR4/MyD88/NF‐κB molecules and NF‐κB activation indices was assessed. In parallel, an IL‐1β–stimulated primary chondrocyte model was used to assess JDJM effects on inflammatory responses, apoptosis, and viability, thereby strengthening mechanistic consistency across systems. In addition, exploratory fecal 16S rRNA sequencing was performed to evaluate microbiota changes after JDJM treatment and their correlations with inflammatory indices, providing testable leads for gut–joint axis–related mechanisms.

## 2 Materials and Methods

### 2.1 Drug Preparation and Quality Control

JDJM, used in the present study, was provided by the Pharmacy Department of the First Affiliated Hospital of Hunan University of Chinese Medicine (Preparation Approval No. Z20190064; Batch No. 20191111). Its prescription included *Achyranthes bidentata* (6 g), *Eucommia ulmoides* (12 g), *Taxillus chinensis* (18 g), *Poria cocos* (12 g), *Asarum heterotropoides* (3 g), *Angelica pubescens* (6 g), *Gentiana macrophylla* (6 g), *Codonopsis pilosula* (12 g), *Cinnamomum cassia* (3 g), *Saposhnikovia divaricata* (6 g), *Paeonia lactiflora* (10 g), *Ligusticum chuanxiong* (6 g), *Glycyrrhiza uralensis* (3 g), prepared *Rehmannia glutinosa* (15 g), *Angelica sinensis* (12 g), *Chaenomeles speciosa* (12 g), *Scutellaria baicalensis* (6 g), *Clematis chinensis* (12 g), and processed *Arisaema erubescens* (6 g). Glucosamine sulfate capsules were used as a reference positive treatment and were supplied by Zhejiang Hisun Pharmaceutical Co. Ltd. (Batch No. 71812133; Changsha, China).

#### 2.1.1 Preparation Process

The herbal materials were soaked in distilled water at a 10:1 (v/v) water‐to‐herb ratio for 30 min and then decocted at 100°C for 120 min. After filtration, the residue was subjected to a second extraction with distilled water at eightfold herb volume for 60 min. The two filtrates were combined and concentrated under reduced pressure at 60°C to a relative density of 1.050. Sodium benzoate (2.5 g) was added, and distilled water was used to make up the final volume to 1000 mL. The extract was then aseptically dispensed into 250 mL bottles, yielding a final crude drug concentration of 0.664 g/mL.

#### 2.1.2 Quality Control

To ensure batch‐to‐batch consistency and traceability, a previously published chromatography–mass spectrometry quality‐control protocol was used as the release criterion. In previous work, a total ion chromatogram fingerprint for JDJM was established using UPLC–Q‐TOF–MS, and inter‐batch consistency was evaluated. High similarity among batch fingerprints was observed, indicating a stable preparation process. In addition, representative constituents were qualitatively identified through database searches, literature comparison, and confirmation with reference standards, generating a component profile [21]. Because this quality‐control system has been published, chromatograms/mass spectra and detailed parameters are not repeated here. All JDJM batches used in this study were prepared using the same process and quality standards and were released according to these criteria.

### 2.2 Main Experimental Reagents and Equipment

The primary antibodies applied in this study were directed against TLR2 (66645‐1‐Ig), TLR4 (19811‐1‐AP), MyD88 (23230‐1‐AP), NF‐κB (10745‐1‐AP), and β‐actin (60008‐1‐Ig), all of which were purchased from ProteinTech Group Inc. (Chicago, IL, USA). The NF‐κB p65 antibody (ab16502), the rabbit anti‐type II collagen antibody (ab34712), and toluidine blue were obtained from Abcam (Cambridge, UK). Antibodies against IL‐6 (bs‐6312R), IL‐1β (bs‐0812R), IFN‐γ (bs‐0480r), and TNF‐α (bsm33207m) were supplied by Beijing Bioss Co. Ltd. (Beijing, China). Primers were synthesized by Sangon Biotech Co. Ltd. (Shanghai, China), and their detailed sequences are listed in Table 1. Reagents used for H&E staining were purchased from Wellbio Co. (Catalog Numbers K435960 and K484350), whereas the immunofluorescence staining reagents were obtained from ProteinTech (Catalog No. SA00013‐2). The Annexin V‐FITC Apoptosis Detection Kit (KGA108) was sourced from KeyGen Biotech (Nanjing, China), TRIzol reagent (15596026) was obtained from Thermo Fisher Scientific (Waltham, MA, USA), and the ECL detection reagent (K‐12045‐D50) was purchased from Advansta (San Jose, CA, USA). An optical microscope (BA410T) was provided by Motic China Group Co. Ltd. (Xiamen, China), while the microplate reader (DSZ2000X) was acquired from Zhongxian Hengye Corp. (Beijing, China). In addition, the BCA protein quantification kit (AWB0104) was purchased from Abiowell (China), and chemiluminescence imaging was performed using the ChemiScope 6100 system from Clinx (Guangzhou, China).

**Table 1:** Sequences of primers used in the qRT‐PCR.

Gene	Primer	Sequence of primers (5′–3′)	Product size/bp
TLR4	Forward	AGAAATCTGGGAGCCCTGTG	151
	Reverse	GCTATGGCTGCCTAAATGCTC	
MyD88	Forward	CCCTTTGTCTCTCGACTCTTGG	185
	Reverse	TACGAGAACAGCCACTGCCC	
NF‐κB1	Forward	ATGCCAATGCCCTCTTCGACT	164
	Reverse	CGTGACTTCCAGCAGATCCCT	
TLR2	Forward	CCTGCTGACGCTGAAAAACC	101
	Reverse	TCAGCCGTCTCAACCTTTCC	
Actin	Forward	TGGCCGAGGACTTTGATTGT	170
	Reverse	TTACACAAATGCGATGCTGCC	

### 2.3 Animals and Model Establishment

#### 2.3.1 Animals and Ethical Approval

In total, 36 male New Zealand White rabbits, aged 3 months and weighing 2.0–2.5 kg, were obtained from the Animal Laboratory of Hunan University of Chinese Medicine (Animal License No. SCXK [Hunan] 2020–0005). Each rabbit was maintained in an individually ventilated cage under standardized environmental conditions, including a temperature of 22°C ± 2°C, relative humidity of 55°C ± 5%, and a 12 h light/dark schedule. The animals were kept in a certified facility (Institutional License No. SYXK [Hunan] 20190009) and were allowed unrestricted access to food and water throughout the study. All experimental procedures involving animals received approval from the Institutional Ethics Committee (Approval No. LLBH‐202007070001) and were conducted in compliance with applicable guidelines for animal care and welfare.

#### 2.3.2 Group Allocation and Randomization

The group size of six rabbits was determined based on our previous experimental experience and a previously published study using the same modified Videman rabbit KOA model and a comparable experimental design [22]. In the previous study, six rabbits were allocated to each experimental group. Based on the OARSI data from that study, a conservative sample‐size estimation assuming a two‐sided *α* of 0.05 and 80% statistical power indicated a minimum requirement of 5 animals/group. After a 2‐week acclimatization period, rabbits were randomly divided into a blank control (BC) group (*n* = 6) and a modeling group (*n* = 30). After 6 weeks of model induction, the modeled animals were randomly allocated by random‐number table into five groups (*n* = 6/group): model control (MC), JDJM low‐dose (LD), JDJM medium‐dose (MD), JDJM high‐dose (HD), and positive control (PC).

#### 2.3.3 KOA Model Establishment

Based on previous studies and the modeling experience of our research group, a modified Videman method was selected to replicate the KOA model. This involved immobilization of the knee joint using a plaster cast for 6 weeks, a technique in which our group has demonstrated proficiency [22].

#### 2.3.4 Drug Administration

After model establishment, rabbits received JDJM by oral gavage at the indicated doses. Doses were calculated using body surface area–based human‐to‐animal conversion, as described in Pharmacological Experimental Methodology [23]. The converted human‐equivalent dose was defined as LD; MD and HD were set at 3× and 9× LD, respectively. JDJM was administered at 3.88, 11.64, and 34.92 g/kg·day to the LD, MD, and HD groups, respectively. The PC group received GS at 100 mg/kg·day, whereas the BC and MC groups received normal saline by gavage (20 mL/kg·day). Treatments were administered twice daily for 6 consecutive weeks.

#### 2.3.5 Sample Collection

Upon completion of the drug administration period, blood, knee joint, and fecal samples were collected for subsequent detection of relevant indicators. The modeled‐side knee joints were harvested for pathological staining. Subsamples were stored in cryotubes at −80°C for quantitative real‐time polymerase chain reaction (qRT‐PCR) and western blot (WB) analyses. Euthanasia was performed on individual animals in accordance with the IAEC guidelines for animal experimentation.

#### 2.3.6 Blinding and Exclusion Criteria

Histological scoring, WB band quantification, and immunofluorescence image analysis were performed by researchers blinded to group allocation. Predefined exclusion criteria included modeling failure, severe infection or skin necrosis, and death unrelated to the experimental intervention. No animals met these exclusion criteria during the study. All 36 rabbits completed the experimental protocol and were included in the final analysis; therefore, no animals were excluded or lost during the experiment.

### 2.4 Cell Culture

Primary rabbit knee‐joint chondrocytes were isolated and cultured in Dulbecco's modified Eagle's medium supplemented with 10% fetal bovine serum. Cells were passaged after trypsinization, and adherent cells were collected for subculture. Third‐passage (P3) chondrocytes were randomized into nine groups and treated with JDJM at 0, 50, 100, 200, 400, 800, 1000, 1600, or 2000 ng/μL to identify an appropriate working concentration. Chondrocytes were stimulated with IL‐1β (10 ng/mL) to establish an in vitro inflammatory model and were cultured on coverslips for subsequent fixation with 4% paraformaldehyde. Toluidine blue staining and type II collagen immunocytochemistry were performed to confirm chondrocyte identity. P3 chondrocytes were then randomized into five groups. Except for the control group, cells were pretreated with IL‐1β for 24 h to induce inflammatory injury. After IL‐1β was removed, cells were treated with JDJM (0, 100, 200, or 400 ng/μL) for 24 h. These concentrations were selected based on the dose‐screening results [24].

### 2.5 Cytotoxicity and Apoptosis Assays

The cytotoxic effect of JDJM on P3 rabbit chondrocytes was evaluated with a CCK‐8 assay. Cells were plated in 96‐well culture plates at a density of 1 × 10^4^ cells/well and exposed to IL‐1β together with different concentrations of JDJM for 24 h. After treatment, 20 μL of CCK‐8 solution was added to each well, and the cells were further incubated for 4 h at 37°C in a humidified atmosphere containing 5% CO_2_. Optical density was subsequently determined at 450 nm using a microplate reader.

Apoptosis in P3 chondrocytes following treatment with IL‐1β (10 ng/mL) and JDJM was analyzed by flow cytometry. The cells were dissociated using 0.25% trypsin supplemented with 0.02% EDTA, harvested by centrifugation at 1500 rpm for 5 min, and rinsed with PBS. They were then stained with Annexin V‐FITC and propidium iodide, and the proportion of apoptotic cells was measured with an Annexin V‐FITC apoptosis detection kit in accordance with the manufacturer's protocol.

### 2.6 Histology and Immunofluorescence

Tissue specimens were first deparaffinized with xylene, followed by rehydration in a series of graded ethanol solutions and rinsing in distilled water. For hematoxylin and eosin staining, the sections were treated sequentially with hematoxylin and eosin, dehydrated through ascending concentrations of ethanol (95%–100%), mounted, and then observed under a light microscope. For immunofluorescence analysis, antigen retrieval was performed using heat treatment in EDTA buffer (pH 9.0). After the slides had cooled to room temperature, they were washed with 0.01 mol/L PBS (pH 7.4–7.6), incubated with Sudan Black to quench tissue autofluorescence, and washed again. The sections were subsequently blocked and incubated with a primary antibody against NF‐κB p65, followed by exposure to a fluorescently labeled secondary antibody. Nuclear counterstaining was carried out with DAPI at 37°C in accordance with the manufacturer's instructions. Finally, the slides were mounted and examined under a fluorescence microscope to evaluate the expression of NF‐κB p65 and its translocation into the nucleus.

### 2.7 Model Validation and Histological Assessment

Model establishment was validated by gross examination and histological analysis. After sectioning, cartilage tissues were stained with H&E, and OA severity was assessed at 100× and 400× magnification using the OARSI scoring system [25]. Two blinded observers scored the sections independently, and the mean value was used for statistical analysis.

### 2.8 Cytokine Determination

Serum levels of IL‐1β, IL‐6, TNF‐α, and IFN‐γ, as well as IL‐1β, IL‐6, and TNF‐α concentrations in cell culture supernatants, were determined by ELISA following the manufacturer's instructions. Each biological sample was measured using at least three technical replicates, and the mean of the technical replicates was used for subsequent statistical analysis.

### 2.9 Western Blot Analysis

For the animal study, approximately 0.025 g of cartilage was harvested for whole‐protein isolation, while cellular protein extracts for the in vitro assays were obtained from chondrocytes after the indicated treatments. All specimens were maintained on ice, rinsed with prechilled PBS, and lysed in 300 μL of RIPA lysis buffer. The resulting extracts were combined with sample loading buffer, heated at 95°C–100°C for 5 min for denaturation, and then immediately placed on ice. Protein levels were quantified using a BCA protein assay kit. Equivalent amounts of protein were subsequently resolved by SDS–PAGE for 130 min. After electrophoresis, the membranes were blocked with 5% skim milk for 1 h at room temperature and then incubated with primary antibodies overnight at 4°C. Following incubation with HRP‐linked secondary antibodies, protein bands were visualized with an ECL detection reagent and captured using a chemiluminescence imaging system.

### 2.10 Quantitative Real‐Time Polymerase Chain Reaction

RNA was extracted using TRIzol reagent in accordance with the manufacturer's instructions. Complementary DNA was then synthesized from the isolated RNA with a reverse transcription kit (CW2141; Beijing ComWin Biotech Co. Ltd., China). The synthesized cDNA was used as the template for quantitative PCR, and each sample was assayed in triplicate. The expression of TLR2, TLR4, MyD88, and NF‐κB was normalized to β‐actin, and relative transcript levels were calculated by the 2^−ΔΔCt^ method [26].

### 2.11 16S rRNA Gene Amplicon Sequencing and Bioinformatic Analysis

Genomic DNA was isolated with a commercial extraction kit following the supplier's protocol. DNA integrity and quantity were evaluated by agarose gel electrophoresis together with microvolume spectrophotometry. Samples meeting quality requirements were used for PCR amplification of the bacterial 16S rRNA V3–V4 hypervariable region with primers 341F (CCTACGGGNGGCWGCAG) and 805R (GACTACHVGGGTATCTAATCC), after which sequencing libraries were prepared. Libraries that passed quality control were sequenced on the Illumina NovaSeq platform using a paired‐end 250 bp strategy to obtain raw sequence data. The raw reads were subsequently filtered to eliminate adapter contamination and low‐quality bases, and chimeras were discarded to produce clean reads for subsequent analyses. Sequence processing and downstream microbiome analyses were conducted in QIIME 2. Denoising was carried out with DADA2, which included error modeling, correction, and chimera detection, to generate amplicon sequence variants (ASVs). Each ASV was then taxonomically classified to produce annotation results and an ASV count table. Using the ASV abundance matrix, relative taxonomic abundance and α‐diversity metrics were calculated. Shared and group‐specific ASVs were displayed with Venn diagrams and petal plots. Community composition and between‐sample dissimilarity were further characterized through PCoA, PCA, NMDS, and hierarchical clustering analyses. Differences in overall microbial community structure among groups were assessed by PERMANOVA (Adonis) and ANOSIM, whereas α‐diversity comparisons were performed using nonparametric statistical methods. Differentially abundant taxa were screened with Wald tests. Additional downstream analyses included LEfSe, functional inference based on PICRUSt2, and visualization with Krona.

### 2.12 Molecular Docking Analysis

Molecular docking was performed to explore the potential interactions between the 21 identified constituents of JDJM and key proteins associated with the TLR4/MyD88/NF‐κB signaling pathway, including the TLR4/MD‐2 complex, MyD88, and NF‐κB p65. The three‐dimensional structures of the target proteins were obtained from the Protein Data Bank (https://www.rcsb.org/), and ligand structures were obtained from PubChem (https://pubchem.ncbi.nlm.nih.gov/). Using PyMOL software, small‐molecule ligands and water molecules that bind to the target protein were removed. The optimized structure was then imported into AutoDock Tools for hydrogenation and molecular docking; molecular docking was performed using AutoDock Vina, and the resulting ligand–target conformation was visualized using PyMOL software.

### 2.13 Outcome Measures

The primary histopathological assessment comprised complementary evaluation of cartilage morphology by H&E staining and quantitative assessment using the OARSI histological score, with the OARSI score serving as the quantitative primary endpoint. Secondary mechanistic outcomes included serum concentrations of IL‐1β, IL‐6, TNF‐α, and IFN‐γ, cartilage expression of TLR4, MyD88, and NF‐κB at the protein and/or transcript levels, and NF‐κB p65 nuclear translocation. TLR2 expression was considered an additional supportive mechanistic measure rather than a core component of the predefined TLR4/MyD88/NF‐κB mechanistic axis.

The in vitro experiments provided supportive mechanistic evidence and included chondrocyte viability, apoptosis, inflammatory cytokine release, and expression of TLR2/TLR4/MyD88‐related molecules. Gut microbiota composition, diversity, differential taxa, and microbiota–phenotype correlation analyses were considered exploratory outcomes.

### 2.14 Statistical Analysis

Data are presented as mean ± standard deviation or median with interquartile range, according to their distribution. Before inferential analyses, the normality of continuous data was assessed using the Shapiro–Wilk test, and homogeneity of variances was evaluated using Levene's test. For normally distributed data with homogeneous variances, comparisons between two groups were performed using the independent‐samples *t*‐test, whereas comparisons among more than two groups were performed using one‐way analysis of variance (ANOVA), followed by Tukey's multiple‐comparisons test. For data that did not meet the assumptions of parametric testing, comparisons between two groups were performed using the Mann–Whitney *U* test, and comparisons among multiple groups were performed using the Kruskal–Wallis test followed by Dunn's multiple‐comparisons test. Categorical data, where applicable, were analyzed using the chi‐square test or Fisher's exact test. Correlations between gut microbiota features and inflammatory or pathway‐related variables were assessed using Spearman's rank correlation analysis. For all experiments, *n* represents independent biological or experimental replicates rather than technical replicates. Technical replicates were averaged before statistical analysis. The exact *n* for each analysis is specified in the corresponding figure legends.

Statistical analyses were performed using SPSS version 26.0, and figures were generated using GraphPad Prism version 10.0. All statistical tests were two‐tailed, and *p* < 0.05 was considered statistically significant. For key pairwise comparisons, between‐group effect estimates and corresponding 95% confidence intervals (CIs) were reported where applicable.

## 3 Results

### 3.1 In Vivo Experiments

#### 3.1.1 JDJM Ameliorated KOA and Alleviated the Inflammatory Response in KOA

A rabbit KOA model was established using a modified Videman method. After 6 weeks of model establishment, animals received treatments by oral gavage according to group assignment. After 6 weeks of treatment, animals were euthanized, and the modeled (left) knee joints were harvested for histological evaluation. H&E‐stained sections showed more severe joint damage and cartilage thinning in the MC group than in the BC group. In contrast, cartilage morphology was improved in the JDJM‐treated groups relative to the MC group (Figure 1A). Consistently, OARSI scores were higher in the MC group than in the BC group, whereas all JDJM dose groups showed lower OARSI scores than the MC group (Figure 1B).

To assess KOA‐associated inflammation and the anti‐inflammatory effects of JDJM, analyses were performed on blood and cartilage samples. Serum and cartilage levels of IL‐1β, IL‐6, TNF‐α, and IFN‐γ were quantified (Figure 1C). Compared with the BC group, inflammatory markers were elevated in the MC group (IL‐1β: Δ = 19.59, 95% CI 14.87–24.31; IL‐6: Δ = 77.58, 95% CI 67.85–87.32; TNF‐α: Δ = 0.3337, 95% CI 0.1897–0.4776; IFN‐γ: Δ = 44.58, 95% CI 35.06–54.10; all *p* < 0.001), indicating systemic inflammatory activation in KOA. Compared with the MC group, inflammatory markers were reduced in all JDJM dose groups, with more consistent reductions in the medium‐ and high‐dose groups. In the HD group, IL‐1β (Δ = −18.65, 95% CI −23.37 to −13.93, *p* < 0.001), IL‐6 (Δ = −54.63, 95% CI −64.36 to −44.89, *p* < 0.001), TNF‐α (Δ = −0.3093, 95% CI −0.4533 to −0.1654, *p* < 0.001), and IFN‐γ (Δ = −32.72, 95% CI −42.24 to −23.21, *p* < 0.001) were reduced. In the MD group, IL‐1β (Δ = −18.14, 95% CI −22.86 to −13.42, *p* < 0.001), IL‐6 (Δ = −43.84, 95% CI −53.58 to −34.11, *p* < 0.001), TNF‐α (Δ = −0.1720, 95% CI −0.3160 to −0.02805, *p* = 0.0141), and IFN‐γ (Δ = −20.19, 95% CI −29.71 to −10.68, *p* < 0.001) were reduced. In the LD group, IL‐1β (Δ = −15.06, 95% CI − 19.79 to −10.34, *p* < 0.001), IL‐6 (Δ = −31.87, 95% CI −41.60 to −22.13, *p* < 0.001), and IFN‐γ (Δ = −10.14, 95% CI −19.66 to −0.6183, *p* = 0.0331) were reduced, whereas the difference in TNF‐α between the LD and MC groups was not significant (*p* = 0.0781). Collectively, JDJM attenuated histological cartilage damage and reduced circulating pro‐inflammatory cytokines in KOA rabbits, supporting a dose‐dependent anti‐inflammatory and chondroprotective effect.

**Figure 1:**
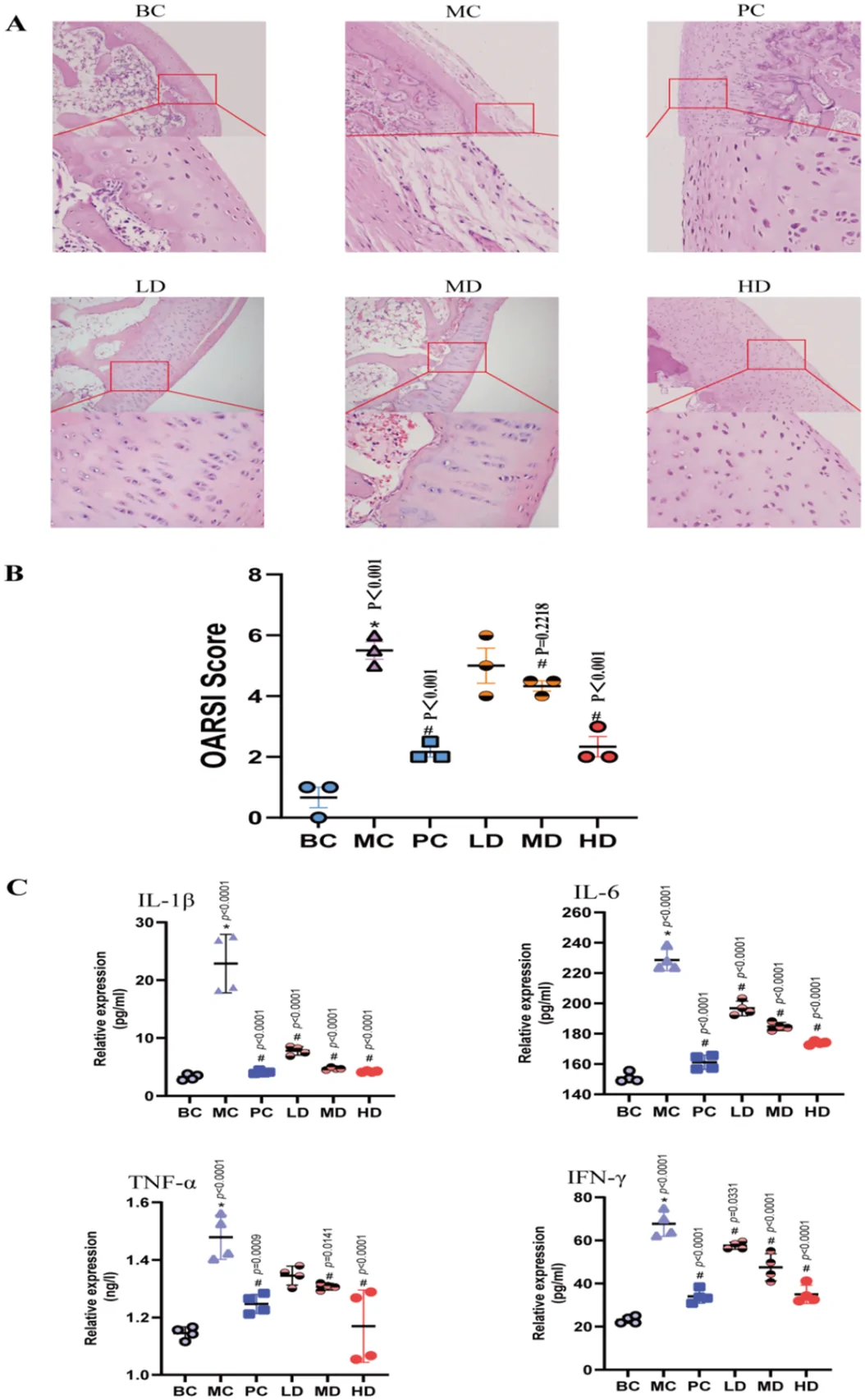
Effects of JDJM on KOA rabbits. (A) Representative hematoxylin and eosin (H&E) images of articular cartilage from the modeled knee joint and (B) OARSI scores derived from H&E‐stained sections (*n* = 3 animals/group). Images were acquired at 100× and 400×; the boxed region indicates the higher‐magnification view. (C) Serum levels of IL‐1β, IL‐6, TNF‐α, and IFN‐γ were measured by ELISA (*n* = 4 animals/group). Group comparisons were performed using one‐way ANOVA followed by Tukey's multiple‐comparisons test. Data are presented as individual data points with the mean and 95% CI. *p < 0.05 versus BC; ^#^ *p* < 0.05 versus MC. Exact *p* values for pairwise comparisons are provided.

#### 3.1.2 JDJM Alleviates Rabbit Knee Osteoarthritis by Inhibiting the TLR4/MyD88/NF‐κB Signaling Pathway

To clarify how JDJM exerts its anti‐inflammatory effects, we measured the expression of TLR4, MyD88, and NF‐κB at both the protein and transcript levels in articular cartilage and further examined the nuclear translocation of NF‐κB p65. Overall, the TLR4/MyD88/NF‐κB pathway was markedly activated in the KOA model. As shown in Figure 2A,B, protein analysis demonstrated that, relative to the BC group, the MC group exhibited significantly higher levels of TLR4, MyD88, and NF‐κB (TLR4: Δ = 0.6012, 95% CI 0.3401–0.8623; MyD88: Δ = 0.5356, 95% CI 0.2308–0.8404; NF‐κB: Δ = 0.5924, 95% CI 0.4768–0.7080; all *p* < 0.001). Following JDJM administration, these protein levels declined compared with those in the MC group, with the most pronounced decreases observed in the HD group, including TLR4 (Δ = −0.3751, 95% CI −0.6366 to −0.1137, *p* = 0.005), MyD88 (Δ = −0.3332, 95% CI −0.6380 to −0.0285, *p* = 0.029), and NF‐κB (Δ = −0.3874, 95% CI −0.5039 to −0.2709, *p* < 0.001). NF‐κB protein expression was also significantly lowered in the LD group (Δ = −0.1276, 95% CI −0.2437 to −0.0115, *p* = 0.028) and the MD group (Δ = −0.2361, 95% CI −0.3524 to −0.1198, *p* < 0.001), whereas the reductions in TLR4 and MyD88 at these two doses were not statistically significant.

**Figure 2:**
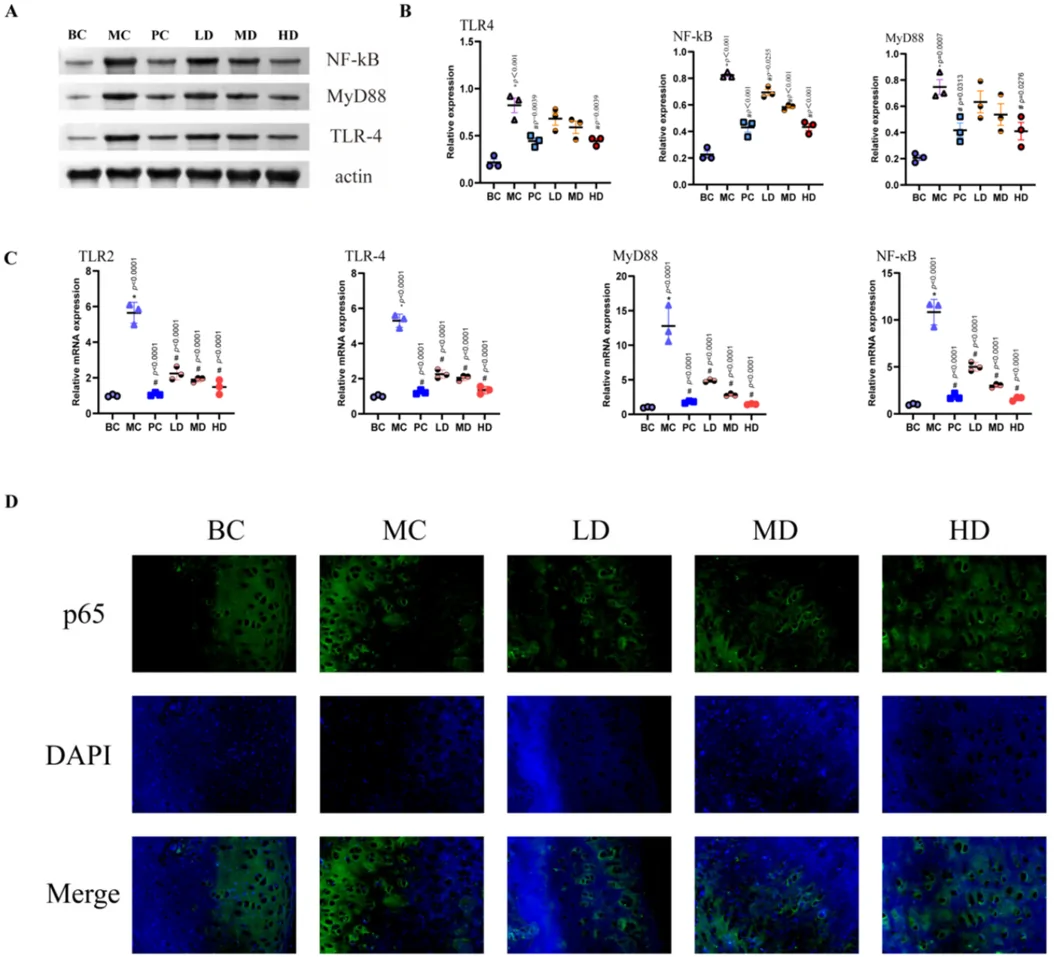
JDJM alleviates rabbit KOA by suppressing the TLR4/MyD88/NF‐κB signaling cascade. (A and B) Protein expression of TLR4, MyD88, and NF‐κB was determined by Western blotting, with β‐actin used as the loading control (*n* = 3 animals/group). (C) The mRNA levels of TLR2, TLR4, MyD88, and NF‐κB were measured by RT‐qPCR (*n* = 3 animals/group). (D) Representative immunofluorescence images of NF‐κB p65 intracellular distribution were obtained from *n* = 2 animals/group at 400× magnification; these images were used for qualitative assessment only and were not subjected to inferential statistical analysis. Each quantitative data point represents an individual animal. Data are presented as mean with 95% CI. Differences among groups were analyzed by one‐way ANOVA followed by Tukey's multiple‐comparisons test. *p < 0.05 versus BC; ^#^ *p* < 0.05 versus MC. Exact *p* values for the specified pairwise comparisons are provided.

To verify these observations, we next evaluated mRNA expression of TLR2, TLR4, MyD88, and NF‐κB in cartilage tissue (Figure 2C). In comparison with the BC group, the MC group showed markedly elevated transcript levels for all four genes (TLR2: Δ = 4.650, 95% CI 3.737–5.564; TLR4: Δ = 4.298, 95% CI 3.696–4.899; MyD88: Δ = 11.79, 95% CI 8.738–14.850; NF‐κB: Δ = 9.835, 95% CI 8.157–11.510; all *p* < 0.001), which paralleled the protein findings. Compared with the MC group, the PC group displayed reduced mRNA expression, and a similar downward trend was detected across all JDJM‐treated groups. The strongest suppression again appeared in the HD group, with decreases in TLR2 (Δ = −4.166, 95% CI −5.080 to −3.253, *p* < 0.001), TLR4 (Δ = −3.950, 95% CI −4.551 to −3.349, *p* < 0.001), MyD88 (Δ = −11.33, 95% CI −14.390 to −8.277, *p* < 0.001), and NF‐κB (Δ = −9.224, 95% CI −10.90 to −7.547, *p* < 0.001). By contrast, the LD group showed the smallest, although still significant, reductions (TLR2: Δ = −3.408, 95% CI −4.321 to −2.494, *p* < 0.001; TLR4: Δ = −3.040, 95% CI −3.642 to −2.439, *p* < 0.001; MyD88: Δ = −7.946, 95% CI −11.000 to −4.891, *p* < 0.001; NF‐κB: Δ = −5.829, 95% CI −7.507 to −4.151, *p* < 0.001). Immunofluorescence staining further supported these results. In the BC group, NF‐κB p65 was mainly distributed in the cytoplasm, whereas the MC group exhibited enhanced nuclear accumulation. Treatment with JDJM markedly decreased p65 nuclear localization (Figure 2D), indicating reduced activation of NF‐κB signaling.

Taken together, these findings indicate that the TLR4/MyD88/NF‐κB pathway is abnormally activated in KOA cartilage and that JDJM effectively suppresses pathway‐related molecules as well as NF‐κB p65 nuclear translocation. This provides mechanistic support for the anti‐inflammatory and cartilage‐protective actions of JDJM. In addition, the altered expression of TLR2 mRNA implies that other innate immune receptors may also participate in the therapeutic response, although their precise roles remain to be clarified in future studies.

### 3.2 In Vitro Experiments

To further validate the direct effects of JDJM on chondrocytes, an in vitro primary chondrocyte model was established.

#### 3.2.1 JDJM Enhances the Viability of Rabbit Primary Chondrocytes Within a Defined Concentration Range

To assess the effects of JDJM on rabbit primary chondrocytes, P3 chondrocytes were treated with graded JDJM concentrations, and viability was quantified by CCK‐8. The results (Figure 3A) showed the highest viability at 400 ng/μL. Viability decreased at concentrations > 400 ng/μL. Accordingly, 400 ng/μL was selected for subsequent experiments, and dose‐response analyses were performed at 0, 100, 200, and 400 ng/μL.

**Figure 3:**
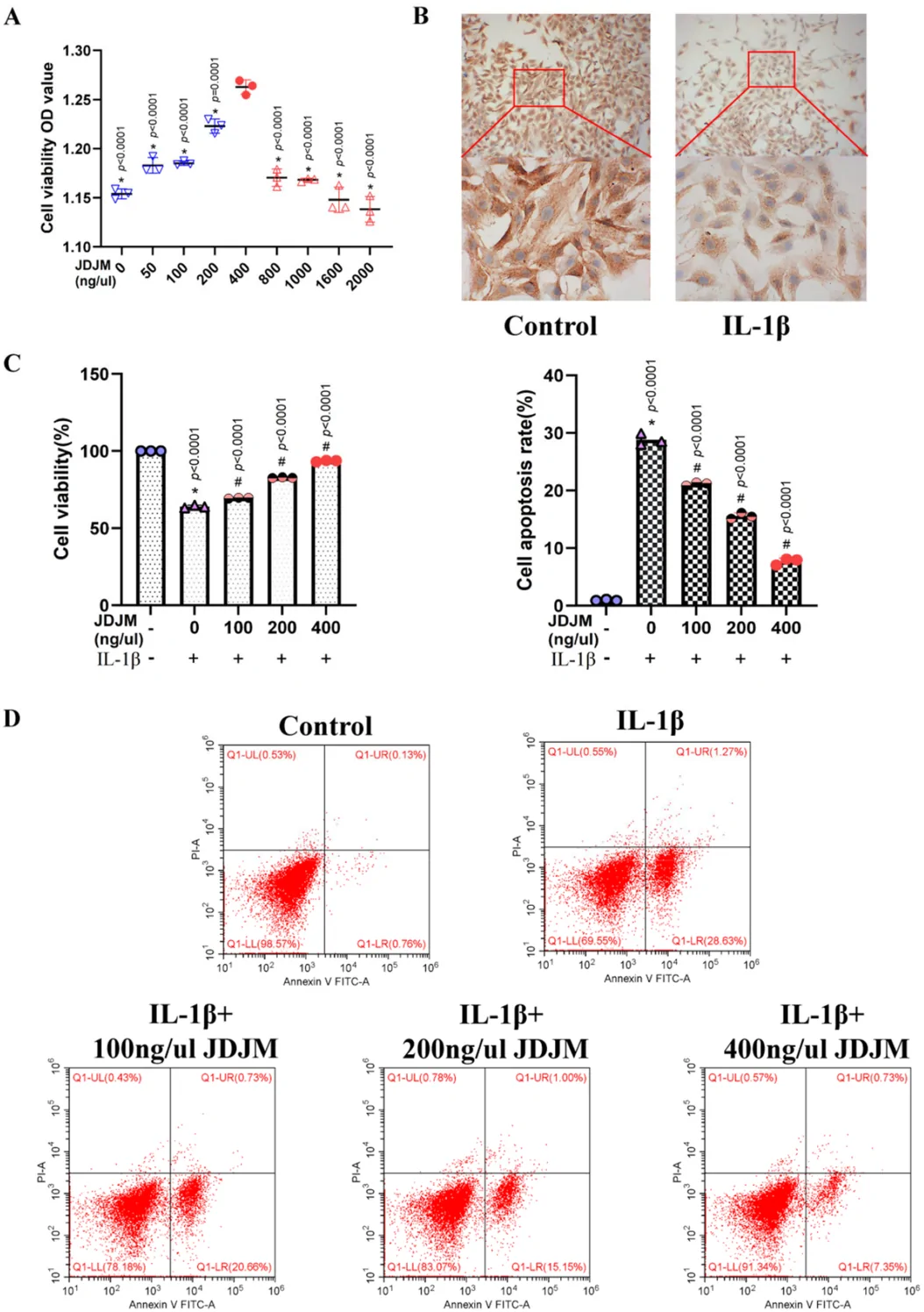
JDJM improves the viability of primary rabbit chondrocytes and attenuates IL‐1β–induced inflammatory injury. (A) Cell viability of primary rabbit chondrocytes treated with increasing concentrations of JDJM. *p < 0.05 versus the 400 ng/μL group. (B) Representative images of type II collagen (COL2A1) staining in control and IL‐1β‐treated chondrocytes at 100× and 400× magnification. (C) Schematic illustration of the experimental design. Primary chondrocytes were exposed to IL‐1β (10 ng/mL) for 24 h, followed by treatment with JDJM (0, 100, 200, or 400 ng/μL) for an additional 24 h after IL‐1β removal. The control group was defined as JDJM−/IL‐1β− and the IL‐1β group as JDJM−/IL‐1β+. (D) Chondrocyte apoptosis was assessed by flow cytometry. Viability and apoptosis were normalized to the control group (100%). Quantitative data are presented as individual data points with the mean and 95% CI. *n* = 3 independent experiments, with at least three technical replicates per experiment; technical replicates were averaged before statistical analysis. *p < 0.05 versus control; ^#^ *p* < 0.05 versus IL‐1β. Exact *p* values for the relevant pairwise comparisons are indicated.

#### 3.2.2 Establishment and Validation of an IL‐1β–Induced Chondrocyte Inflammatory Injury Model

Chondrocytes were treated with IL‐1β for 24 h to induce inflammatory injury, and type II collagen expression was used to validate model establishment. As shown in Figure 3B, collagen II expression was reduced in the IL‐1β–treated group relative to controls, consistent with successful model establishment [27].

#### 3.2.3 JDJM Attenuates IL‐1β–Induced Chondrocyte Injury, Apoptosis, and Inflammatory Mediator Release

Chondrocytes were treated with JDJM, and viability was assessed. The results (Figure 3C,D) showed higher viability (Δ = 35.99, 95% CI 34.72–37.26, *p* < 0.001) and lower apoptosis (Δ = −27.86, 95% CI −29.39 to −26.32, *p* < 0.001) in controls than in the IL‐1β group, indicating that IL‐1β induced chondrocyte injury and apoptosis. JDJM treatment increased viability and reduced apoptosis in IL‐1β–injured chondrocytes. The strongest effects were observed at 400 ng/μL (vs. IL‐1β: viability Δ = 29.58, 95% CI 28.31–30.85, *p* < 0.001; apoptosis Δ = −21.12, 95% CI −22.65 to −19.58, *p* < 0.001), consistent with the dose‐screening results.

Inflammatory cytokines in culture supernatants were quantified. As shown in Figure 4D, cytokine levels were higher in the IL‐1β group than in controls (IL‐1β: Δ = 51.53, 95% CI 46.86–56.19, *p* < 0.001; IL‐6: Δ = 36.16, 95% CI 31.31–41.02, *p* < 0.001; TNF‐α: Δ = 93.83, 95% CI 83.62–104.00, *p* < 0.001). JDJM reduced these cytokines, with the largest reductions at 400 ng/μL (IL‐1β: Δ = −34.00, 95% CI −38.67 to −29.33, *p* < 0.001; IL‐6: Δ = −28.07, 95% CI −32.92 to −23.21, *p* < 0.001; TNF‐α: Δ = −67.02, 95% CI −77.23 to −56.81, *p* < 0.001). Collectively, JDJM attenuated IL‐1β–induced inflammatory injury in primary chondrocytes in vitro, as evidenced by increased viability, reduced apoptosis, and decreased cytokine release, consistent with the in vivo findings.

**Figure 4:**
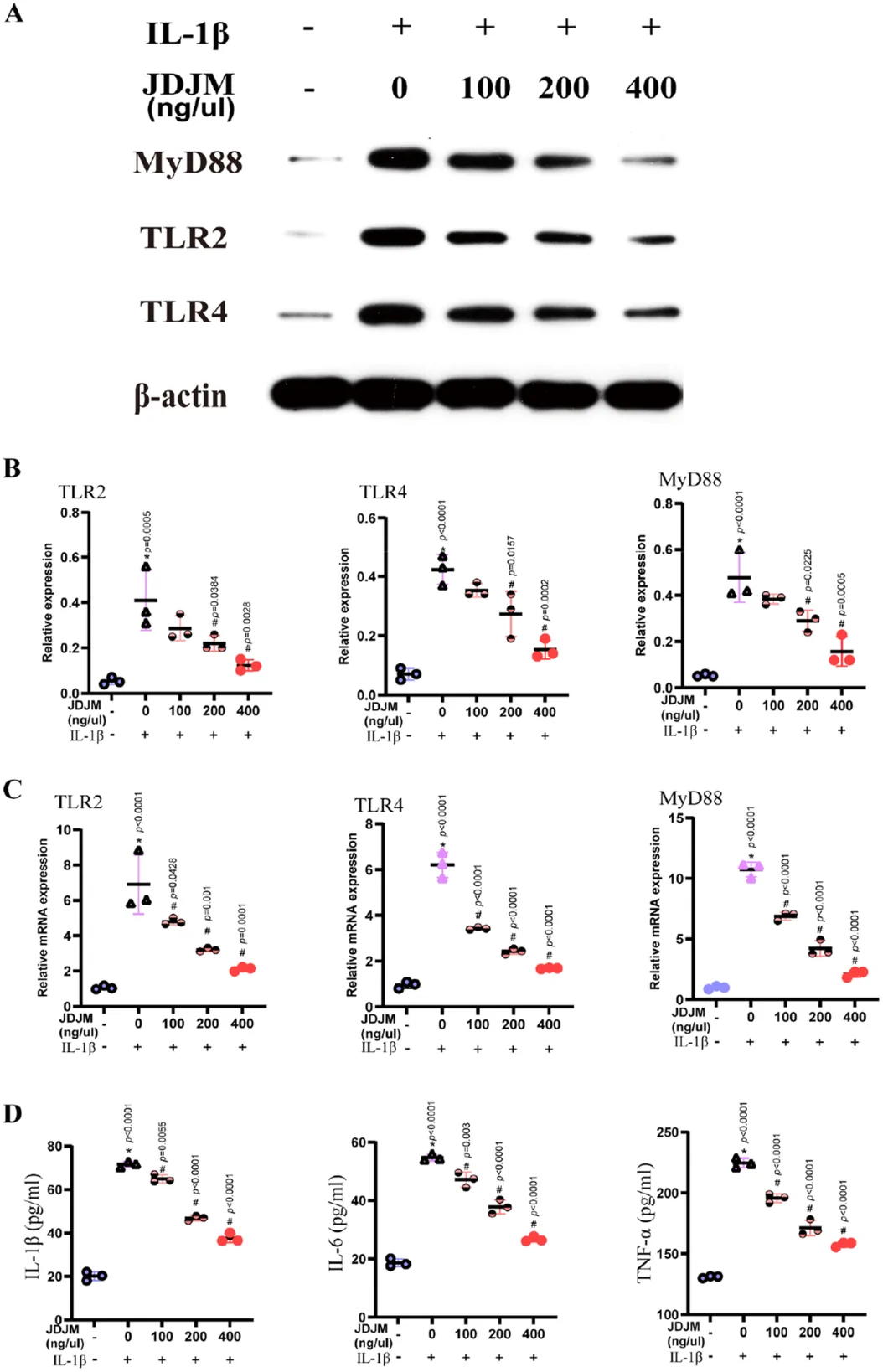
JDJM mitigates IL‐1β–induced inflammatory injury in chondrocytes by suppressing TLR2/TLR4/MyD88 signaling. (A and B) Western blotting was performed to detect TLR2, TLR4, and MyD88 protein expression, with β‐actin serving as the loading control. (C) RT‐qPCR was used to assess the mRNA expression of TLR2, TLR4, and MyD88. (D) Levels of IL‐1β, IL‐6, and TNF‐α in culture supernatants were measured by ELISA. *n* = 3 independent experiments, with at least three technical replicates per experiment. Technical replicates were averaged before statistical analysis. Differences among groups were analyzed using one‐way ANOVA followed by Tukey's multiple‐comparisons test. Data are presented as individual data points with the mean and 95% CI. *p < 0.05 versus the control group; ^#^ *p* < 0.05 versus the IL‐1β group. Exact *p* values for the relevant pairwise comparisons are indicated.

#### 3.2.4 JDJM Attenuates IL‐1β–Induced Chondrocyte Inflammatory Injury by Inhibiting TLR2/TLR4/MyD88 Signaling

To further elucidate the anti‐inflammatory mechanism of JDJM in chondrocytes, protein and mRNA levels of TLR2, TLR4, and MyD88 were assessed in IL‐1β–stimulated primary chondrocytes in vitro. Western blotting (Figure 4A,B) showed higher TLR2, TLR4, and MyD88 protein levels in the IL‐1β group than in controls (TLR2: Δ = 0.3567, 95% CI 0.1760–0.5373, *p* = 0.0005; TLR4: Δ = 0.3533, 95% CI 0.2312–0.4755, *p* < 0.001; MyD88: Δ = 0.4233, 95% CI 0.2620–0.5847, *p* < 0.001). JDJM reduced these proteins in a dose‐dependent manner, with the largest reductions observed at 400 ng/μL (vs. IL‐1β: TLR2 Δ = −0.2867, 95% CI −0.4673 to −0.1060, *p* = 0.0028; TLR4 Δ = −0.2700, 95% CI −0.3922 to −0.1478, *p* = 0.0002; MyD88 Δ = −0.3200, 95% CI −0.4814 to −0.1586, *p* = 0.0005).

RT‐qPCR results were consistent with the protein findings (Figure 4C). Compared with controls, mRNA levels of TLR2, TLR4, and MyD88 were higher in the IL‐1β group (TLR2: Δ = 5.856, 95% CI 3.807–7.904, *p* < 0.001; TLR4: Δ = 5.249, 95% CI 4.539–5.960, *p* < 0.001; MyD88: Δ = 9.762, 95% CI 8.567–10.96, *p* < 0.001). JDJM reduced these transcripts, with the largest reductions at 400 ng/μL (vs. IL‐1β: TLR2 Δ = −4.794, 95% CI −6.843 to −2.746, *p* = 0.0001; TLR4 Δ = −4.522, 95% CI −5.233 to −3.811, *p* < 0.001; MyD88 Δ = −8.627, 95% CI −9.821 to −7.432, *p* < 0.001). Reductions were smaller at lower JDJM concentrations.

Collectively, JDJM reduced TLR2, TLR4, and MyD88 at both the protein and mRNA levels in the IL‐1β–induced chondrocyte inflammatory injury model. These molecular changes were consistent with improved viability, reduced apoptosis, and decreased cytokine release.

### 3.3 Molecular Docking Analysis of JDJM Constituents With TLR4/MyD88/NF‐κB Pathway Proteins

Molecular docking was performed to evaluate the potential interactions between 21 identified constituents of JDJM and 3 key proteins associated with the TLR4/MyD88/NF‐κB signaling pathway. As shown in Figure 5A, the tested constituents exhibited distinct docking‐score profiles against the TLR4/MD‐2 complex, MyD88, and NF‐κB p65. Following refined docking of the five highest‐ranked compounds for each target, wogonoside showed the most favorable docking score against the TLR4/MD‐2 complex (−8.417 kcal/mol), baicalin against MyD88 (−7.919 kcal/mol), and glycyrrhizic acid against NF‐κB p65 (−8.573 kcal/mol).

**Figure 5:**
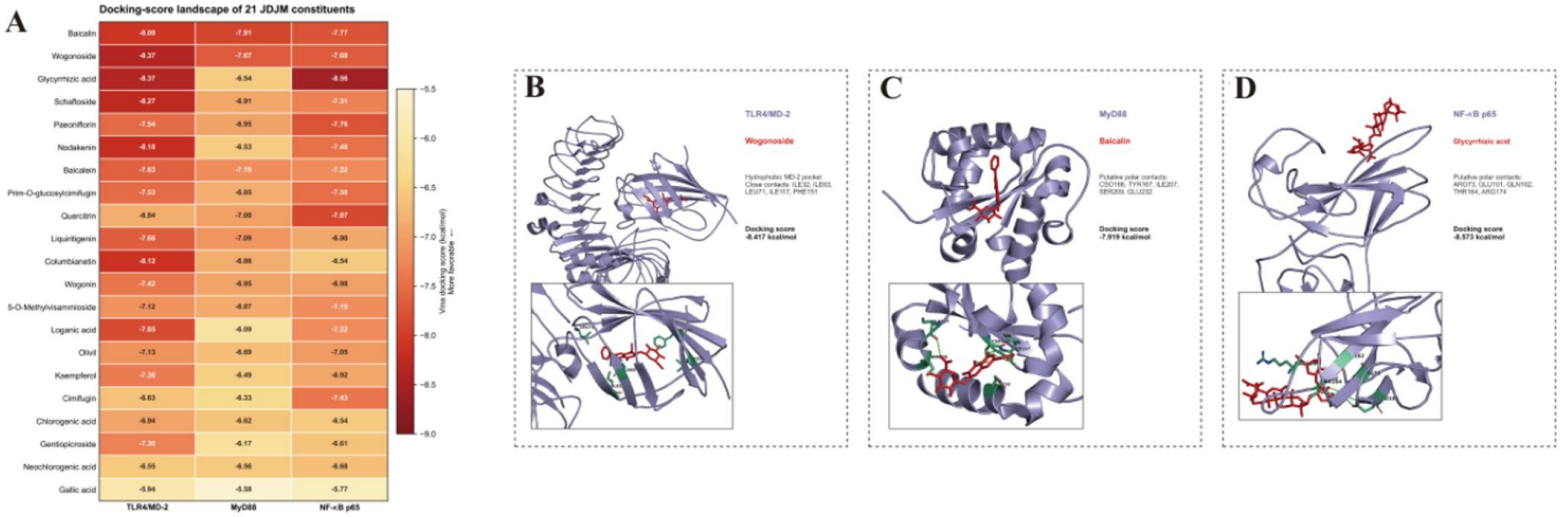
Molecular docking of JDJM constituents with proteins associated with the TLR4/MyD88/NF‐κB signaling pathway. (A) Heatmap of AutoDock Vina scores for 21 identified JDJM constituents against the TLR4/MD‐2 complex, MyD88, and NF‐κB p65. More negative values indicate more favorable predicted docking poses within the same target. (B–D) Representative docking poses of wogonoside with TLR4/MD‐2 (−8.417 kcal/mol), baicalin with MyD88 (−7.919 kcal/mol), and glycyrrhizic acid with NF‐κB p65 (−8.573 kcal/mol), respectively. Green dashed lines indicate predicted polar contacts.

Representative binding poses showed that wogonoside was accommodated within the MD‐2 pocket and interacted with residues including Ile32, Ile63, Leu71, Ile117, and Phe151 (Figure 5B). Baicalin showed predicted polar contacts with CSO166, Tyr167, Ile207, Ser209, and Glu232 of MyD88 (Figure 5C), whereas glycyrrhizic acid interacted with Arg73, Glu101, Gln162, Thr164, and Arg174 of NF‐κB p65 (Figure 5D). These findings provide supportive computational evidence for potential interactions between multiple JDJM constituents and proteins associated with the TLR4/MyD88/NF‐κB pathway.

### 3.4 Alterations in Gut Microbiota of KOA Rabbit Models and the Intervention Effects of JDJM

To evaluate associations between KOA induction, JDJM treatment, and gut microbiota changes, fecal samples were subjected to 16S rRNA gene amplicon sequencing, and an ASV feature table was generated using DADA2. The Venn diagram (Figure 6B) showed that only 38 ASVs were shared across the 6 groups, suggesting group‐level differences in microbiota composition. The BC group contained 1831 unique ASVs, whereas the MC group contained 1543. After JDJM treatment, the HD group contained 1316 unique ASVs and showed larger shifts in community structure than the MD and LD groups. Collectively, KOA induction and JDJM treatment were associated with changes in gut microbiota composition.

**Figure 6:**
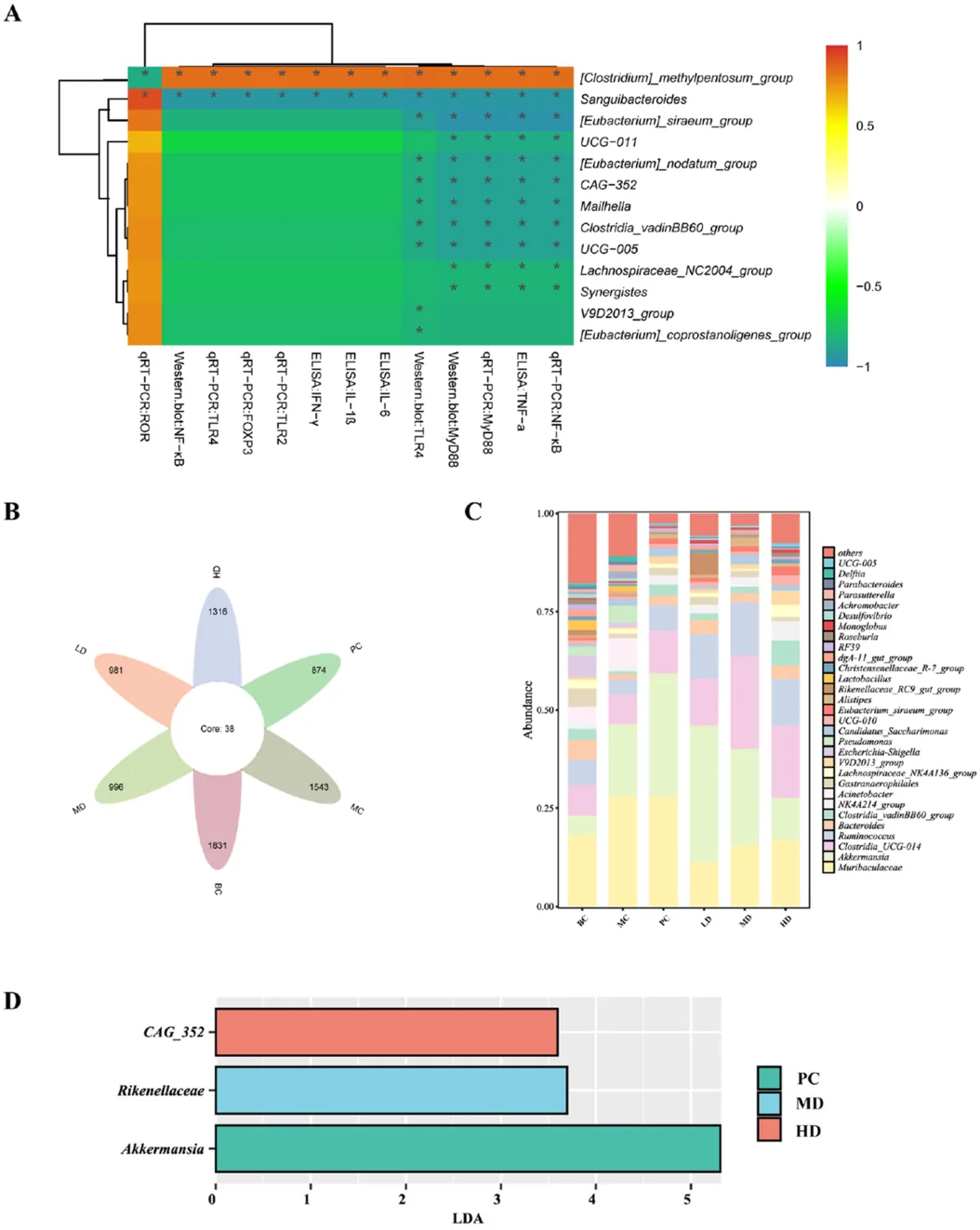
Gut microbiota alterations in KOA rabbits and the effects of JDJM treatment. (A) Spearman's correlation analysis between gut microbiota features and inflammatory indicators and TLR4/MyD88/NF‐κB‐related molecules. (B) Venn diagram showing shared and unique amplicon sequence variants (ASVs) across groups. (C) Genus‐level taxonomic composition based on relative abundance. (D) LEfSe analysis identifying differentially abundant taxa across groups. 16S rRNA gene sequencing was performed using *n* = 3 animals/group, with each sample derived from an individual animal.

Genus‐level profiling (Figure 6C) indicated a shift in the MC group relative to the BC group, with higher relative abundances of the Gram‐negative genera *Acinetobacter* (6.89%) and *Pseudomonas* (4.32%) [28, 29]. In parallel, *Ruminococcus* (3.94%) and *Bacteroides* (1.40%) showed lower relative abundances in the MC group. After JDJM treatment, the HD group showed reduced *Acinetobacter* and *Pseudomonas* (to near/below the detection limit) and an increased *Ruminococcus* relative abundance (11.83%). In addition, *Akkermansia*, which was elevated in the MC group, decreased to 10.83% in the HD group. These results suggest that JDJM—particularly at the high dose—was associated with KOA‐related genus‐level shifts, aligning with the anti‐inflammatory and chondroprotective phenotypes observed in vivo.

To identify taxa distinguishing groups, LEfSe analysis was performed (Figure 6D). The PC group was characterized by enrichment of *Akkermansia* (LDA = 5.31, *p* < 0.05), the MD group by Rikenellaceae (LDA = 3.70, *p* < 0.05), and the HD group by CAG_352 (LDA = 3.60, *p* < 0.05). These findings suggest dose‐associated differences in discriminative taxa after JDJM treatment; however, a formal dose–response relationship would require dedicated trend testing.

To explore links between microbiota composition and inflammatory/pathway readouts, Spearman's correlation analysis was performed (Figure 6A). *Sanguibacteroides* abundance was negatively correlated with pro‐inflammatory cytokines and key TLR4/MyD88/NF‐κB pathway proteins, whereas the *Clostridium methylpentosum* group showed positive correlations with these inflammatory readouts and pathway proteins. The *Eubacterium siraeum* group was negatively correlated with NF‐κB and MyD88 mRNA levels measured by RT‐qPCR. Overall, gut microbiota features correlated with inflammatory indicators and pathway activation measures, consistent with the overall effects observed after JDJM treatment. However, because causal approaches (e.g., antibiotic depletion or fecal microbiota transplantation) were not performed, these correlations cannot establish causality and require further validation.

## 4 Discussion

As an age‐related degenerative joint disease, KOA is currently managed primarily with lifestyle modification, exercise‐based rehabilitation, and analgesic/anti‐inflammatory drugs [30]. However, long‐term pharmacotherapy carries gastrointestinal and renal risks and rarely reverses disease progression, creating a major unmet need for interventions that combine safety with structural benefit [31]. In this study, the effects of JDJM in KOA were evaluated using parallel in vivo and in vitro (primary chondrocyte) models. The main findings were as follows: (1) in a rabbit KOA model established using a modified Videman method, JDJM improved articular cartilage histopathology and reduced OARSI scores, supporting a potential structural benefit; (2) JDJM reduced circulating inflammatory cytokines in vivo and, in an IL‐1β–induced inflammatory injury model in primary chondrocytes in vitro, increased viability, reduced apoptosis, and decreased cytokine release; and (3) JDJM treatment was accompanied by shifts in gut microbiota composition, and several genera correlated with inflammatory markers and pathway‐related molecules, suggesting a potential gut–joint link. Collectively, these results suggest that JDJM may improve inflammation‐related readouts and confer phenotype‐level benefits by attenuating key steps in inflammatory signal amplification in KOA.

KOA pathogenesis involves more than mechanical wear; it reflects imbalanced remodeling across multiple tissues and cell types, in which chronic low‐grade inflammation contributes to cartilage degeneration and pain [3, 32]. Inflammatory stimuli can shift chondrocytes from a matrix‐maintaining state toward a catabolic, pro‐inflammatory phenotype, promoting cytokine release, extracellular matrix degradation, and cell death, and amplifying inflammatory crosstalk with synovial and immune cells [33, 34, 35]. Cytokines such as IL‐1β, TNF‐α, and IL‐6 function as both effectors and amplifiers, promoting catabolic programs and accelerating cartilage degeneration [36, 37]. In vivo, elevated pro‐inflammatory cytokines accompanied cartilage structural damage in the model group, and these cytokines were reduced after JDJM treatment. In primary chondrocytes in vitro, JDJM reduced IL‐1β–induced apoptosis and cytokine release. Relative to prior work emphasizing correlations between inflammation and cartilage damage, the present study linked reduced inflammatory cytokines with concomitant improvements in histopathology and cellular phenotypes, strengthening the association between anti‐inflammatory effects and cartilage protection. In addition, the dose‐related improvement in inflammatory markers and injury phenotypes supports the pharmacodynamic plausibility of the findings [38, 39].

Mechanistically, TLR‐mediated innate immune sensing and NF‐κB–driven transcription are considered key axes of inflammatory amplification in KOA [40]. TLR4 responds to DAMPs and microbial cues, activates MyD88‐dependent signaling, and promotes NF‐κB activation, thereby upregulating pro‐inflammatory genes (e.g., IL‐1β, TNF‐α, IL‐6) and reinforcing a feed‐forward loop of receptor activation, transcriptional induction, inflammatory amplification, and tissue injury [11]. NF‐κB p65 nuclear translocation is a key cellular marker of functional pathway activation and can be more informative than measurements of total protein or mRNA alone [41]. In vivo, TLR4/MyD88/NF‐κB upregulation accompanied increased p65 nuclear localization in the model group, whereas JDJM treatment reduced pathway components and decreased p65 nuclear localization. In primary chondrocytes in vitro, JDJM attenuated IL‐1β–induced upregulation of TLR4/MyD88‐related molecules, consistent with reduced apoptosis and cytokine release. Compared with studies limited to a single model or readout, this work integrates histopathology, inflammatory cytokines, and pathway activation markers, strengthening the linkage between pathway inhibition and structural/cellular benefits. Notably, TLR2 changes were also observed at the mRNA and protein levels, suggesting additional involvement of innate immune receptors. However, the relative contributions of TLR2 and TLR4 may vary across models and tissues. Future studies should delineate the respective roles of TLR2 and TLR4 and distinguish MyD88‐dependent from MyD88‐independent signaling in refined experimental systems.

Molecular docking further supported the potential involvement of JDJM constituents in TLR4/MyD88/NF‐κB signaling. Wogonoside, baicalin, and glycyrrhizic acid showed favorable predicted interactions with TLR4/MD‐2, MyD88, and NF‐κB p65, respectively. These findings provide complementary computational support for the experimental results but require further validation of direct target engagement. The gut microbiota findings should be interpreted as exploratory. 16S rRNA sequencing indicated that KOA induction was associated with altered microbiota composition. After JDJM treatment, community composition shifted, and several genera correlated with inflammatory cytokines and TLR4/MyD88/NF‐κB–related molecules, providing clues for a potential gut–joint link in KOA. However, correlation does not establish causality: microbiota changes may occur secondary to reduced systemic inflammation, or they may modulate TLR4 signaling through metabolites, intestinal barrier function, and endotoxin exposure [42]. Because causal validation (e.g., antibiotic depletion or fecal microbiota transplantation) was not performed, these results should be viewed as hypothesis‐generating. Future studies could integrate FMT or depletion–reconstitution designs with measurements of serum LPS, intestinal barrier markers, and joint immune cell infiltration to define causal contributions and mediating mechanisms.

Several limitations of this study should be acknowledged. First, although consistent changes in inflammatory phenotypes and TLR4/MyD88/NF‐κB‐related readouts were observed in both the animal and cellular experiments, pathway‐specific pharmacological or genetic perturbation and rescue experiments were not performed. Therefore, the present findings support the involvement of this signaling axis but do not establish its functional necessity for the effects of JDJM. Second, the animal sample size was relatively limited, and functional outcomes such as pain‐related behavior, gait, and joint mobility were not systematically assessed. In addition, only male rabbits were included, and potential sex‐related differences in disease progression and treatment response could therefore not be evaluated. Third, JDJM is a multicomponent herbal preparation. Although molecular docking identified several candidate constituent–target interactions, direct target engagement, pharmacokinetic exposure, and exposure–response relationships were not examined. Finally, the gut microbiota analysis was exploratory and based on a limited number of samples. The observed microbiota alterations and their associations with inflammatory or pathway‐related measures therefore should not be interpreted as evidence of a causal microbiota‐mediated mechanism. Antibiotic depletion, fecal microbiota transplantation, or other microbiota‐directed interventions were not performed in the present study.

Future studies should address these unresolved questions at several levels. At the signaling level, pathway‐specific pharmacological inhibition or activation, gene silencing, and rescue experiments will be required to determine the functional contribution of TLR4/MyD88/NF‐κB signaling to the anti‐inflammatory and chondroprotective effects of JDJM. At the systemic level, antibiotic depletion and fecal microbiota transplantation, combined with assessment of intestinal barrier integrity, circulating LPS‐related indices, and microbial metabolites, may help determine whether gut microbiota alterations actively contribute to the joint response or primarily accompany changes in systemic inflammation. At the pharmacological level, further studies integrating quantitative chemical profiling, pharmacokinetic analysis, and experimental validation of candidate constituent–target interactions may help identify the components most relevant to the observed biological effects. Finally, prospective clinical studies using standardized JDJM preparations will be needed to determine whether the experimental findings translate into improvements in pain, joint function, and structural outcomes in patients with KOA.

## 5 Conclusion

This study showed that JDJM attenuated cartilage histopathology and reduced inflammatory readouts in a rabbit KOA model and an IL‐1β–induced chondrocyte inflammatory injury model. Mechanistically, JDJM inhibited TLR4/MyD88/NF‐κB signaling and reduced NF‐κB p65 nuclear translocation, with concomitant reductions in chondrocyte apoptosis and inflammatory injury. 16S rRNA sequencing suggested that these effects were accompanied by shifts in gut microbiota composition; however, causality requires validation in dedicated experiments (e.g., antibiotic depletion or fecal microbiota transplantation).

## Author Contributions

**Jin Wu:** conceptualization, methodology, writing – original draft, writing – review and editing. **Zhi Wen:** conceptualization, methodology, writing – review and editing, writing – original draft. **Zifeng Ye:** investigation, formal analysis, data curation. **Liguo Qiu:** investigation, formal analysis, data curation. **Guang Xia:** validation, writing – review and editing. **Xuyi Tan:** resources, writing – review and editing, supervision. **Tikang Guo:** validation, writing – review and editing. **Yong Tang:** validation, writing – review and editing. **Min Lu:** resources, writing – review and editing, supervision. **Xiaotong Xu:** resources, writing – review and editing, supervision. **Gaoyan Kuang:** resources, writing – review and editing, supervision.

## Ethics Statement

The protocols for conducting animal experiments were approved by the Ethics Committee of Hunan University of Chinese Medicine (LLBH‐202007070001) and were conducted in accordance with the National Institutes of Health Guide for the Care and Use of Laboratory Animals.

## Consent

The authors have nothing to report.

## Conflicts of Interest

The authors declare no conflicts of interest.

## Supporting information


Supporting File

## Data Availability

The data that support the findings of this study are available from the corresponding author upon reasonable request.

## References

[1] D. J. Hunter , “Lower Extremity Osteoarthritis Management Needs a Paradigm Shift,” British Journal of Sports Medicine 45, no. 4 (2011): 283–288, 10.1136/bjsm.2010.081117. 21297174

[2] A. Wojcieszek , A. Kurowska , A. Majda , H. Liszka , and A. Gądek , “The Impact of Chronic Pain, Stiffness and Difficulties in Performing Daily Activities on the Quality of Life of Older Patients With Knee Osteoarthritis,” International Journal of Environmental Research and Public Health 19, no. 24 (2022): 16815, 10.3390/ijerph192416815. PMC9779661 36554695

[3] Q. Yao , X. Wu , C. Tao , et al., “Osteoarthritis: Pathogenic Signaling Pathways and Therapeutic Targets,” Signal Transduction and Targeted Therapy 8, no. 1 (2023): 56, 10.1038/s41392-023-01330-w. PMC9898571 36737426

[4] M. Porter and J. M. Wilkinson , “Patient Safety After Partial and Total Knee Replacement,” Lancet 384, no. 9960 (2014): 2106, 10.1016/S0140-6736(14)62365-5. 25497192

[5] W. Glinkowski , D. Śladowski , W. Tomaszewski , et al., “Molecular Mechanisms and Therapeutic Role of Intra‐Articular Hyaluronic Acid in Osteoarthritis: A Precision Medicine Perspective,” Journal of Clinical Medicine 14, no. 8 (2025): 2547, 10.3390/jcm14082547. PMC12027770 40283379

[6] C. D. King , K. T. Sibille , B. R. Goodin , et al., “Experimental Pain Sensitivity Differs as a Function of Clinical Pain Severity in Symptomatic Knee Osteoarthritis,” Osteoarthritis and Cartilage 21, no. 9 (2013): 1243–1252, 10.1016/j.joca.2013.05.015. PMC3831366 23973137

[7] Q. Wang , K. Onuma , C. Liu , et al., “Dysregulated Integrin αVβ3 and CD47 Signaling Promotes Joint Inflammation, Cartilage Breakdown, and Progression of Osteoarthritis,” JCI Insight 4, no. 18 (2019): e128616, 10.1172/jci.insight.128616. PMC6795293 31534047

[8] S. K. Satyanarayanan and B. K. Rajendran , “Editorial: Unveiling Inflammaging‐Mechanistic Insights on Aging and Related Diseases,” Frontiers in Medicine 12 (2025): 1607050, 10.3389/fmed.2025.1607050. PMC12052547 40330777

[9] C. Sun , X. Zhou , T. Guo , and J. Meng , “The Immune Role of the Intestinal Microbiome in Knee Osteoarthritis: A Review of the Possible Mechanisms and Therapies,” Frontiers in Immunology 14 (2023): 1168818, 10.3389/fimmu.2023.1168818. PMC10306395 37388748

[10] T. Yamada and A. Takaoka , “Innate Immune Recognition Against SARS‐CoV‐2,” Inflammation and Regeneration 43, no. 1 (2023): 7, 10.1186/s41232-023-00259-5. PMC9879261 36703213

[11] X. Liu , B. Lu , J. Fu , X. Zhu , E. Song , and Y. Song , “Amorphous Silica Nanoparticles Induce Inflammation via Activation of NLRP3 Inflammasome and HMGB1/TLR4/MYD88/NF‐kb Signaling Pathway in HUVEC Cells,” Journal of Hazardous Materials 404, no. Pt B (2021): 124050, 10.1016/j.jhazmat.2020.124050. 33053467

[12] J. Cheng , R. Zhang , Z. Xu , et al., “Early Glycolytic Reprogramming Controls Microglial Inflammatory Activation,” Journal of Neuroinflammation 18, no. 1 (2021): 129, 10.1186/s12974-021-02187-y. PMC8191212 34107997

[13] Y. Xu , S. Xue , T. Zhang , et al., “Toddalolactone Protects Against Osteoarthritis by Ameliorating Chondrocyte Inflammation and Suppressing Osteoclastogenesis,” Chinese Medicine 17, no. 1 (2022): 18, 10.1186/s13020-022-00576-w. PMC8817519 35123541

[14] K. Tang , X. Xu , M. Lu , et al., “Jiawei Duhuo Jisheng Mixture Mitigates Osteoarthritis Progression in Rabbits by Inhibiting Inflammation: A Network Pharmacology and Experimental Approach,” Combinatorial Chemistry & High Throughput Screening 28, no. 12 (2025): 2107–2131, 10.2174/0113862073298262240705045345. 38988173

[15] Z. F. Ye , Y. W. Yuan , Z. Wen , et al., “Effect of Jiawei Duhuo Jisheng Mixture in Improving Mitophagy to Treat Knee Osteoarthritis Based on PINK1/Parkin Pathway,” Zhongguo Zhong Yao Za Zhi 50, no. 22 (2025): 6391–6399, 10.19540/j.cnki.cjcmm.20250730.401. 41508078

[16] Q. Shi , L. Huang , J. Duan , G. Kuang , M. Lu , and X. Tan , “The Effects of Jiawei Duhuo Jisheng Mixture on Wnt/β‐Catenin Signaling Pathway in the Synovium Inflamed by Knee Osteoarthritis: An In Vitro and In Vivo Experiment,” Journal of Ethnopharmacology 294 (2022): 115363, 10.1016/j.jep.2022.115363. 35551975

[17] Y. Yang , C. Hao , T. Jiao , et al., “Osteoarthritis Treatment via the GLP‐1‐Mediated Gut‐Joint Axis Targets Intestinal FXR Signaling,” Science 388, no. 6742 (2025): eadt0548, 10.1126/science.adt0548. 40179178

[18] T. Wang , Y. Xu , H. Zha , and L. Wu , “The “Gut Microbiota‐Ferroptosis Axis”: A Hypothesis for Osteoarthritis Pathogenesis and Therapeutic Implications,” Frontiers in Microbiology 16 (2025): 1685962, 10.3389/fmicb.2025.1685962. PMC12647078 41311485

[19] P. Plewa , P. Graczyk , K. Figiel , A. Dach , and A. Pawlik , “Gut and Joint Microbiome and Dysbiosis: A New Perspective on the Pathogenesis and Treatment of Osteoarthritis,” Pathogens 15, no. 1 (2026): 62, 10.3390/pathogens15010062. PMC12845289 41599046

[20] C. M. Park and H. S. Yoon , “Chlorogenic Acid as a Positive Regulator in LPS‐PG‐Induced Inflammation via TLR4/MyD88‐Mediated NF‐κB and PI3K/MAPK Signaling Cascades in Human Gingival Fibroblasts,” Mediators of Inflammation 2022 (2022): 1–8, 10.1155/2022/2127642. PMC9013303 35437426

[21] N. Yi , Mechanistic Study on the Treatment of Knee Osteoarthritis by Jiawei Duhuo Jisheng Mixture and Its Active Components via Regulation of Cartilage Degeneration and Synovial Inflammation (Hunan University of Chinese Medicine, 2023), https://link.cnki.net/doi/10.27138/d.cnki.ghuzc.2023.000006.

[22] X. Xu , N. Li , Y. Wu , et al., “Zhuifeng Tougu Capsules Inhibit the TLR4/MyD88/NF‐κB Signaling Pathway and Alleviate Knee Osteoarthritis: In Vitro and In Vivo Experiments,” Frontiers in Pharmacology 13 (2022): 951860, 10.3389/fphar.2022.951860. PMC9521277 36188596

[23] Y. Li , W. Wei , and X. Wu , Pharmacological Experimental Methodology (People's Medical Publishing House, 2010).

[24] M. Kapoor , J. Martel‐Pelletier , D. Lajeunesse , J. P. Pelletier , and H. Fahmi , “Role of Proinflammatory Cytokines in the Pathophysiology of Osteoarthritis,” Nature Reviews Rheumatology 7, no. 1 (2011): 33–42, 10.1038/nrrheum.2010.196. 21119608

[25] W. Waldstein , G. Perino , S. L. Gilbert , S. A. Maher , R. Windhager , and F. Boettner , “OARSI Osteoarthritis Cartilage Histopathology Assessment System: A Biomechanical Evaluation in the Human Knee,” Journal of Orthopaedic Research 34, no. 1 (2016): 135–140, 10.1002/jor.23010. 26250350

[26] C. Sandoval , L. Mella , K. Godoy , K. Adeli , and J. Farías , “β‐Carotene Increases Activity of Cytochrome P450 2E1 During Ethanol Consumption,” Antioxidants 11, no. 5 (2022): 1033, 10.3390/antiox11051033. PMC9137679 35624897

[27] J. Xu , Z. Ruan , Z. Guo , et al., “Inhibition of SAT1 Alleviates Chondrocyte Inflammation and Ferroptosis by Repressing ALOX15 Expression and Activating the Nrf2 Pathway,” Bone & Joint Research 13, no. 3 (2024): 110–123, 10.1302/2046-3758.133.BJR-2023-0250.R1. PMC10917474 38447596

[28] S. Qin , W. Xiao , C. Zhou , et al., “ *Pseudomonas aeruginosa*: Pathogenesis, Virulence Factors, Antibiotic Resistance, Interaction With Host, Technology Advances and Emerging Therapeutics,” Signal Transduction and Targeted Therapy 7, no. 1 (2022): 199, 10.1038/s41392-022-01056-1. PMC9233671 35752612

[29] E. Geisinger and R. R. Isberg , “Antibiotic Modulation of Capsular Exopolysaccharide and Virulence in *Acinetobacter baumannii* ,” PLoS Pathogens 11, no. 2 (2015): e1004691, 10.1371/journal.ppat.1004691. PMC4334535 25679516

[30] G. O. Cioroianu , A. Florescu , C. E. Simionescu , T. N. Sas , D. N. Tarniţă , and O. C. Rogoveanu , “The Therapeutic Benefits of NSAIDs and Physical Therapy in Knee Osteoarthritis,” Romanian Journal of Morphology and Embryology 65, no. 2 (2024): 217–224, 10.47162/RJME.65.2.08. PMC11384862 39020536

[31] D. A. Iordan , I. Onu , D. V. Matei , et al., “Effect of Hydrocortisone Ultrasonic Phonophoresis in the Treatment of Knee Osteoarthritis,” Balneo and PRM Research Journal 2023 (2023): 575, 10.12680/balneo.2023.575.

[32] A. Fan , G. Wu , J. Wang , et al., “Inhibition of Fibroblast Activation Protein Ameliorates Cartilage Matrix Degradation and Osteoarthritis Progression,” Bone Research 11, no. 1 (2023): 3, 10.1038/s41413-022-00243-8. PMC9806108 36588124

[33] S. Tang , X. Nie , J. Ruan , Y. Cao , J. Kang , and C. Ding , “Circular RNA circNFKB1 Promotes Osteoarthritis Progression Through Interacting With ENO1 and Sustaining NF‐κB Signaling,” Cell Death & Disease 13, no. 8 (2022): 695, 10.1038/s41419-022-05148-2. PMC9363463 35945200

[34] L. Wang , H. Xu , X. Li , et al., “Cucurbitacin E Reduces IL‐1β‐Induced Inflammation and Cartilage Degeneration by Inhibiting the PI3K/Akt Pathway in Osteoarthritic Chondrocytes,” Journal of Translational Medicine 21, no. 1 (2023): 880, 10.1186/s12967-023-04771-7. PMC10696753 38049841

[35] C. Jia , X. Li , J. Pan , et al., “Silencing of Angiopoietin‐Like Protein 4 (Angptl4) Decreases Inflammation, Extracellular Matrix Degradation, and Apoptosis in Osteoarthritis via the Sirtuin 1/NF‐κB Pathway,” Oxidative Medicine and Cellular Longevity 2022 (2022): 1135827, 10.1155/2022/1135827. PMC9442503 36071864

[36] E. Horváth , Á. Sólyom , J. Székely , E. E. Nagy , and H. Popoviciu , “Inflammatory and Metabolic Signaling Interfaces of the Hypertrophic and Senescent Chondrocyte Phenotypes Associated With Osteoarthritis,” International Journal of Molecular Sciences 24, no. 22 (2023): 16468, 10.3390/ijms242216468. PMC10671750 38003658

[37] M. Segarra‐Queralt , G. Piella , and J. Noailly , “Network‐Based Modelling of Mechano‐Inflammatory Chondrocyte Regulation in Early Osteoarthritis,” Frontiers in Bioengineering and Biotechnology 11 (2023): 1006066, 10.3389/fbioe.2023.1006066. PMC9936426 36815875

[38] Y. Ma , Z. Lu , B. Jia , et al., “DNA Origami as a Nanomedicine for Targeted Rheumatoid Arthritis Therapy Through Reactive Oxygen Species and Nitric Oxide Scavenging,” ACS Nano 16, no. 8 (2022): 12520–12531, 10.1021/acsnano.2c03991. 35917300

[39] R. Zhou , S. Xue , Y. Cheng , et al., “Macrophage Membrane‐Camouflaged Biomimetic Nanoparticles for Rheumatoid Arthritis Treatment via Modulating Macrophage Polarization,” Journal of Nanobiotechnology 22, no. 1 (2024): 578, 10.1186/s12951-024-02822-9. PMC11414146 39300463

[40] J. E. Woodell‐May and S. D. Sommerfeld , “Role of Inflammation and the Immune System in the Progression of Osteoarthritis,” Journal of Orthopaedic Research 38, no. 2 (2020): 253–257, 10.1002/jor.24457. 31469192

[41] Y. Pi , Q. Yuan , S. Qin , et al., “ *Chlorella pyrenoidosa* Polysaccharide CPP‐3a Promotes M1 Polarization of Macrophages via TLR4/2‐MyD88‐NF‐κB/p38 MAPK Signaling Pathways,” Marine Drugs 23, no. 7 (2025): 290, 10.3390/md23070290. PMC12299851 40710515

[42] U. G. Longo , A. Lalli , B. Bandini , et al., “Role of the Gut Microbiota in Osteoarthritis, Rheumatoid Arthritis, and Spondylarthritis: An Update on the Gut‐Joint Axis,” International Journal of Molecular Sciences 25, no. 6 (2024): 3242, 10.3390/ijms25063242. PMC10970477 38542216

