# Supplementary material for: Jiawei Duhuo Jisheng Mixture Alleviates Knee Osteoarthritis via Inhibition of TLR4/MyD88/NF‐κB Signaling and Is Associated With Gut Microbiota Alterations
Source: Immun Inflamm Dis. 2026 Nov 12;14(11):e70586. doi: 10.1002/iid3.70586 (PMC13756838; doi:10.1002/iid3.70586)
Supplement: Supplementary file 1 [file IID3-14-e70586-s001.pdf]

NOTE: Please save this file locally before filling in the table, DO NOT work on the file within your internet browser as changes will not be saved. Adobe Acrobat Reader (available free here) is recommended for completion.

The ARRIVE guidelines 2.0: author checklist

The ARRIVE Essential 10

These items are the basic minimum to include in a manuscript. Without this information, readers and reviewers cannot assess the reliability of the findings.

Item		Recommendation	Section/line number, or reason for not reporting
**Study design**	1	For each experiment, provide brief details of study design including:<br>a. The groups being compared, including control groups. If no control group has been used, the rationale should be stated.	Methods, Section 2.3, lines 202 – 206.
		b. The experimental unit (e.g. a single animal, litter, or cage of animals).	Methods, Section 2.14, lines 376 – 379; Figure 2 legend, lines 476 – 482; Figure 6 legend, lines 649 – 650.
**Sample size**	2	a. Specify the exact number of experimental units allocated to each group, and the total number in each experiment. Also indicate the total number of animals used.	Abstract, lines 21 – 24; Methods, Section 2.3, lines 184 – 186 and 202 – 206.
		b. Explain how the sample size was decided. Provide details of any *a priori* sample size calculation, if done.	Methods, Section 2.3, lines 195 – 201.
**Inclusion and exclusion criteria**	3	a. Describe any criteria used for including and excluding animals (or experimental units) during the experiment, and data points during the analysis. Specify if these criteria were established *a priori*. If no criteria were set, state this explicitly.	Methods, Section 2.3, lines 227 – 230.
		b. For each experimental group, report any animals, experimental units or data points not included in the analysis and explain why. If there were no exclusions, state so.	Methods, Section 2.3, lines 230 – 233; Figure legends, lines 421 – 428, 474 – 485, and 644 – 650.
		c. For each analysis, report the exact value of *n* in each experimental group.	Methods, Section 2.14, lines 376 – 379; corresponding figure legends, e.g., lines 421 – 428, 474 – 485, and 644 – 650.
**Randomisation**	4	a. State whether randomisation was used to allocate experimental units to control and treatment groups. If done, provide the method used to generate the randomisation sequence.	Methods, Section 2.3, lines 202 – 206.
		b. Describe the strategy used to minimise potential confounders such as the order of treatments and measurements, or animal/cage location. If confounders were not controlled, state this explicitly.	Not explicitly reported. Animals were randomly allocated to experimental groups as described in lines 202 – 206.
**Blinding**	5	Describe who was aware of the group allocation at the different stages of the experiment (during the allocation, the conduct of the experiment, the outcome assessment, and the data analysis).	Methods, Section 2.3, lines 227 – 229; Section 2.7, lines 277 – 282.
**Outcome measures**	6	a. Clearly define all outcome measures assessed (e.g. cell death, molecular markers, or behavioural changes).	Methods, Section 2.13, lines 347 – 360.
		b. For hypothesis-testing studies, specify the primary outcome measure, i.e. the outcome measure that was used to determine the sample size.	Methods, Section 2.13, lines 347 – 351.
**Statistical methods**	7	a. Provide details of the statistical methods used for each analysis, including software used.	Methods, Section 2.14, lines 362 – 384.
		b. Describe any methods used to assess whether the data met the assumptions of the statistical approach, and what was done if the assumptions were not met.	Methods, Section 2.14, lines 362 – 375.
**Experimental animals**	8	a. Provide species-appropriate details of the animals used, including species, strain and substrain, sex, age or developmental stage, and, if relevant, weight.	Methods, Section 2.3, lines 184 – 186.
		b. Provide further relevant information on the provenance of animals, health/immune status, genetic modification status, genotype, and any previous procedures.	Methods, Section 2.3, lines 185 – 194.
**Experimental procedures**	9	For each experimental group, including controls, describe the procedures in enough detail to allow others to replicate them, including:	Methods, Section 2.3, lines 207 – 226; Sections 2.6 – 2.10, lines 262 – 309.
		a. What was done, how it was done and what was used.	Methods, Section 2.3, lines 201 – 220.
		b. When and how often.	Methods, Section 2.3, lines 184 – 194 and 201 – 202.
		c. Where (including detail of any acclimatisation periods).	
		d. Why (provide rationale for procedures).	Methods, Section 2.3, lines 195 – 220.
**Results**	10	For each experiment conducted, including independent replications, report:<br>a. Summary/descriptive statistics for each experimental group, with a measure of variability where applicable (e.g. mean and SD, or median and range).	Results, Section 3.1, lines 389 – 485; corresponding figure legends, lines 421 – 428 and 474 – 485.
		b. If applicable, the effect size with a confidence interval.	Results, Section 3.1, lines 401 – 416 and 435 – 465; Methods, Section 2.14, lines 382 – 384.
